# Widespread Presence of Domestic Dogs on Sandy Beaches of Southern Chile

**DOI:** 10.3390/ani11010161

**Published:** 2021-01-12

**Authors:** Esteban I. Cortés, Juan G. Navedo, Eduardo A. Silva-Rodríguez

**Affiliations:** 1 Instituto de Conservación, Biodiversidad y Territorio, Facultad de Ciencias Forestales y Recursos Naturales, Universidad Austral de Chile, Valdivia 5110566, Chile; 2 Bird Ecology Lab, Instituto de Ciencias Marinas y Limnológicas, Facultad de Ciencias, Universidad Austral de Chile, Valdivia 5110566, Chile; jgnavedo@uach.cl

**Keywords:** *Canis familiaris*, Chile, dog disturbance, recreation, sandy beaches, shorebirds

## Abstract

**Simple Summary:**

The presence of dogs on sandy beaches is a concern when it comes to protecting the breeding and migratory stop-over sites of shorebirds. To manage this threat, it is necessary to understand the factors that explain the presence of dogs at sandy beaches. To address this, we conducted a study in southern Chile where we surveyed dogs and their tracks at 14 beaches. Dogs were present on all of them, and we found that their abundance was higher when more people were on the beach and at beaches surrounded by more houses. Furthermore, we detected that, given the opportunity, dogs frequently harassed whimbrels, a long-distance migratory shorebird. Dogs at beaches were often not supervised, and only 13% were on a leash. Although our study shows the association between dogs and people, it identifies that this relationship is not simply one of dogs and their owners; instead, it is free-ranging dogs that are associated with beach-goers and human settlements. Therefore, we suggest that to reduce dog harassment of shorebirds, it is fundamental to reduce the number of unsupervised and unleashed dogs at beaches.

**Abstract:**

Dogs on sandy beaches are a threat to shorebirds. Managing this problem requires understanding the factors that influence the abundance of dogs in these ecosystems. We aimed to determine the proportion of beaches used by dogs and the effects of human presence on dog abundance on sandy beaches of southern Chile. We conducted dog counts and recorded the presence of tracks on 14 beaches. We used zero-inflated generalized linear mixed models to determine if the number of people, number of households, and other covariates were associated with dog abundance. We detected dog tracks on all the beaches, and dog sightings on most of them. Dogs were frequently not supervised (45%) and only 13% of them were leashed. The number of people on the beach and the number of houses near the beach were positively associated with the number of dogs on beaches. Finally, when dogs co-occurred with whimbrels (*Numenius phaeopus*), the probability of dog harassment was high (59%). Our work reveals that human presence determines the abundance of dogs on sandy beaches. Therefore, our study suggests that any strategy aiming at reducing dog harassment of shorebirds requires changes in those human behaviors that favor the presence of free-ranging dogs at beaches.

## 1. Introduction

Sandy beaches are one of the most important intertidal ecosystems for wildlife [1], providing a habitat for a great diversity of species, including shorebirds [2]. Sand beaches and dunes also offer key ecosystem services, such as coastal protection, erosion control, and cultural services linked to tourism and recreation [3]. Not surprisingly, beaches are one of the most valued ecosystems for recreational purposes [4], but, paradoxically, this high valuation makes their protection difficult. Intensive recreational use is, in fact, one of the main threats that affect sandy beaches [5]. Recreation can affect the biodiversity of beaches directly, for example, through the use of motorized vehicles [6], or indirectly through measures taken to promote tourism, such as the removal of stranded macroalgae [7] and coastal illumination [8]. In addition, one of the direct consequences of human activity on sandy beaches is the presence of domestic dogs (*Canis familiaris*).

Dogs are the most abundant carnivores globally (ca. 1 billion individuals [9]) and are recognized among the most damaging invasive species worldwide [10], affecting wild animals through predation, disturbance, disease transmission, competition, and hybridization [11]. In the specific case of beaches, dogs prey on some species such as marine turtles’ eggs [12] and shorebird chicks [13] and can be a major source of disturbance for shorebirds [14]. Disturbance involves situations where dogs harass or scare other animals without causing death but leading to behavioral changes and energetic or reproductive costs for the affected individuals [15]. Although in some cases birds can get habituated to disturbance [13] and the population consequences of such disturbance are not clear [16], these events can lead to lower nest attendance [17], temporal decrease in local abundance [18], and lower feeding rates [18,19]. Given that some of these effects can occur even when human–dog abundances are relatively low (e.g., [18]), and that several of these habitat-dependent species show global declining population trends (e.g., [20]), it is important to understand the factors that drive the presence and abundance of dogs on sandy beaches.

One obvious factor that might drive the abundance of dogs on beaches is human presence. People often take dogs to the beaches to allow them to roam freely [21]. For example, dog abundance at beaches can double during weekends [22], when human abundance is higher [23]. Another factor that might explain the presence of dogs on beaches is the proximity to human settlements, as reported for other ecosystems (e.g., [24,25]). Dogs concentrate activity near households [26,27,28] and near the places their owners work [29]. Considering that in many areas of the world dogs are allowed to roam free by their owners (see [9]), it is therefore predictable that in areas with higher human density, dog abundance will be higher.

Within the last decade, a growing number of studies have documented the impacts of dogs on wildlife in Chile (e.g., [24,30,31]). Although most studies have been conducted in forest ecosystems, recent work has reported dog attacks on coastal birds [32] and documented that the combination of dogs and human activity affect the distribution and foraging activity of shorebirds [18]. In Chile, the number of dogs per capita is high (human:dog ratio = 5.2 and 1.7 for urban and rural areas, respectively [9]) and a high proportion of dog owners allow their dogs to roam freely (e.g., >85% in rural areas [33]). This is possibly a consequence of the national-level legislation that is not explicit in mandating the use of leashes for dogs in public spaces [34]. Although there are some local leash regulations (e.g., [35]), these appear to be rarely enforced as adherence is low. The insufficient enforcement of existing regulation, the high proportion of owned dogs allowed to roam [33], and their frequent presence in other ecosystems (e.g., [31]) suggest that their presence at sandy beaches could be generalized to and associated with people.

In this study, we explored the proportion of beaches that are used by dogs to assess the predictors of dog abundance on sandy beach ecosystems in southern Chile. Based on previous studies conducted in other ecosystems (e.g., [24,27]), we predicted that the abundance of dogs will be higher (1) at beaches surrounded by a higher number of households, and (2) at times when the number of visitors is higher. The mechanisms behind the expected patterns are linked to dog ownership. In areas where the number of households is higher, the number of free-ranging owned dogs is also expected to be higher. Similarly, beach visitors often bring dogs; therefore, we expected to detect more dogs at beaches that were used by more people. Finally, we describe dog–shorebird interactions using the whimbrel (*Numenius phaeopus*) as a study model. The results of this study are relevant to inform policy on responsible dog ownership in inhabited areas surrounding sandy beaches.

## 2. Materials and Methods

### 2.1. Study Area

**Figure 1:**
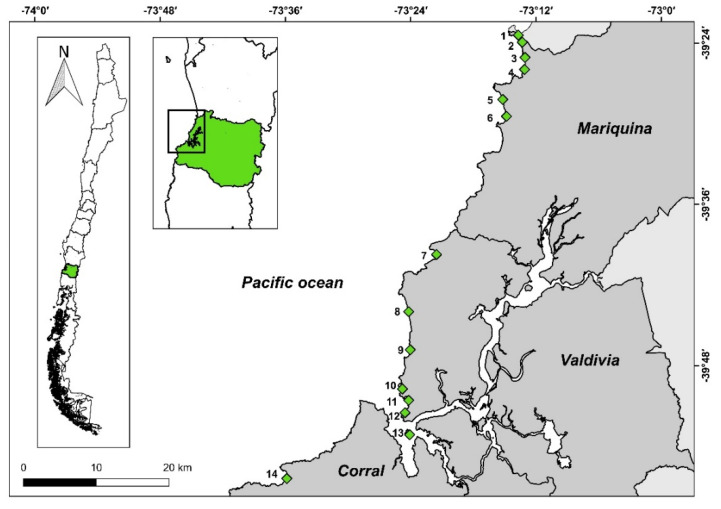
Location of the beaches sampled within the study area: (1) Ronca, (2) Cheuque, (3) Pichicuyín, (4) Mehuín, (5) Epuco, (6) Alepue, (7) Pilolcura, (8) Curiñanco, (9) Calfuco, (10) San Ignacio, (11) Los Molinos, (12) Niebla, (13) Mancera, and (14) Chaihuín. The insets show the location of the study area within Los Ríos region (green) and Chile. Vectorial data were obtained from [39].

The study area is located in the coastal area of Los Ríos region, Chile, between the mouth of the Queule river (39°23′ S, 73°14′ W) and the southern limit of the municipality of Corral (40°0′ S, 73°42′ W). The area includes three municipalities: Mariquina, Valdivia, and Corral (Figure 1). Within the study area, ca. 33 km of the coastline corresponds to sandy beaches. Sandy beaches within the area include reflective, intermediate, and dissipative types [36], although most of them are intermediate [37]. Prior work suggests that there is a low diversity of macroinvertebrates, but some species reach high abundances and are present during the whole year, providing food resources for shorebirds [38]. The area is inhabited by at least nine species of migratory shorebirds, with the whimbrel being one of the most frequent [38].

### 2.2. Sampling Design

We selected 14 sandy beaches (Figure 1). Thirteen of them were exposed to the ocean and a single beach was located on Mancera Island, within the mouth of the Valdivia river (39°53′ S, 73°23′ W). We selected beaches considering logistic issues and avoided sampling beaches located closer than 400 m between them. The mean distance between sampled beaches was 4.8 km (range: 0.4–19.9 km), and the median length of the beaches was 1.2 km. As a whole, we sampled 20.6 km, corresponding to ca. 62% of the total extension of beaches within the study area.

Sampling was conducted during austral summer, between January and March 2020, when recreational activity in this ecosystem is higher. A priori, we expected the abundance of dogs to be associated with the number of people. Therefore, to secure variability in the number of people at beaches, each one was sampled during two different days: once during the week (Friday or Monday), and once during the weekend (Saturday or Sunday). In addition, each beach was sampled twice within each day. The first sampling started before noon (between 8:00 and 12:00), and in all cases finished before 13:30 (Time Zone UTC-3). The second sampling started between 14:00 and 19:30, finishing in all cases before 20:00. The mean time elapsed between the end of the morning and the start of the afternoon sampling was 206 min (range: 60–618 min). This approach was followed on all beaches with a single exception where, due to logistical constraints, the morning sampling corresponding to the weekday was conducted on a different day.

To evaluate dog use of beaches at the site level, we divided each beach into 100 m segments (following [40]). However, segments were on average slightly smaller (mean: 98.3 m; range: 37–100 m), because to cover the full extent of the beach, the length of the last segment was usually smaller. Across all 14 beaches, we surveyed a total of 209 segments. The starting and ending points of each segment were recorded in the field using a GPS (Garmin GPSMAP 64s, Garmin Ltd., Lenexa, KS, USA).

An observer (always the same person, E.C.) walked along the beach from one extreme to the other, recording the number of people and dogs observed within each segment. In addition, in each segment, we recorded the presence of dog tracks as complementary evidence of dog use of the beach. People and dogs were included in our counts if they were detected on the beach below the foredune vegetation (see [1], p. 35). To avoid double counts, individuals were only recorded in the segment where they were first detected. To determine the total number of people and dogs on the beach for each sampling, we summed the counts recorded in each segment of the beach.

For each sampling occasion, we recorded the date, time, and weather (rainy, cloudy, partly cloudy, and sunny). Average walking speed was 2.9 km/h. Dogs recorded were classified in the following categories based on their supervision status at the moment of detection: (1) supervised dogs: if a person was responsible for the dog and it was on a leash or other restriction method; (2) partially supervised dogs: if at least one person appeared to be responsible for the dog, but the dog could roam freely; and (3) unsupervised dogs: if the dog was not under the direct control of a person. Given the high number of stray dogs in Chile [41], it is possible that some of the dogs classified as partially supervised were in fact unsupervised that were in the company of people (but not the owners) during the sampling.

For each beach, we quantified the number of houses located 200 m or less from the beach. This buffer was selected considering that free-ranging owned dogs spend most of their time close to the houses, usually within 200 m [27]. For this purpose, we generated a polygon for each beach considering the borders of the sampling. After this, we generated a 200 m buffer around the polygon, where we quantified the total number of roofs within the area using the images available in GoogleEarth^TM^ in March 2020 (https://www.google.com/intl/es/earth/). We acknowledge that some of the roofs counted were not houses but other buildings.

### 2.3. Dog Harassment of Shorebirds

To determine the probability of dog harassment of shorebirds, we used the whimbrel as a model species. The whimbrel is a good model for this purpose because it is territorial in nonbreeding grounds [42], it is one of the most frequent shorebirds in the study area [38], and there is evidence that similar species are sensitive to disturbance caused by dogs and people (e.g., the Hudsonian godwit, *Limosa haemastica* [18]). During sampling, we recorded all whimbrels detected in the segments and if dogs were present there. We also recorded all the events where dogs were observed harassing whimbrels. Harassment was defined as the persecution of whimbrels by one or more dogs [32]. We did not include the events where whimbrels flew due to the presence of dogs if harassment did not occur. However, we acknowledge that the mere presence of dogs can also lead to nonlethal effects on birds [15,43]. When we detected harassment, we recorded the type of dog involved (supervised, partially supervised, or unsupervised), the number of whimbrels that were chased, the outcome of the harassment (whether birds escaped, were injured, or killed), and, in case of escape, if the birds left the beach or if they landed in another area of the same beach. Birds were observed using 10 × 42 binoculars (Nikon Monarch 3).

### 2.4. Data Analysis

#### 2.4.1. Dog Use of Beaches

To determine the proportion of sandy beaches used by dogs in the study area, we estimated the quotient between the number of beaches where dogs were detected at least once and the total number of beaches. In addition, for each beach, we determined the proportion of segments where we detected dogs at least once considering the four sampling occasions. These metrics were calculated separately for both detection methods used (dog sightings and detection of dog tracks).

#### 2.4.2. Factors that Influence Dog Abundance at Beaches

To determine the factors that explain the abundance of dogs on beaches, we used generalized linear mixed models (GLMMs; [44]). We treated the number of dogs recorded on each beach for each of the sampling occasions (*n* = 56) as the response variable. Considering that multiple sampling occasions within a single beach are unlikely to be independent, we treated “beach identity” as a random effect. In the global model, we included the number of people detected on the beach for each sampling occasion and the number of houses (i.e., the number of roofs) detected within the 200 m buffer as predictor variables. Both variables were log-transformed (log_10_ (number of people + 1), log_10_ (number of houses + 1)). In addition, we included the time of the beginning of the sampling (morning, afternoon) and the day of the sampling (weekday, weekend) as categorical predictors and the length of the beach as a continuous predictor. We note that time and day were included because they were part of the design. Considering that the variance inflation factor was within acceptable limits (VIF < 3, [45]), we kept all variables in the models.

The data contained a relatively large number of zeroes, and during preliminary analyses using Poisson distribution, we detected evidence of overdispersion. Therefore, we used zero-inflated Poisson models for our analyses. This approach allowed us to handle the excess of zeroes and the overdispersion [44]. For the zero-inflated component of the model, we used the number of people on the beach (log_10_ (number of people + 1)) as a predictor. We also considered the option of including weather as a potential covariate; however, rainfall was not independent of the number of people, so we only included the latter.

Models were fitted using the glmmTMB function in package *glmmTMB* [46]. Model selection was conducted using the Akaike Information Criterion corrected for small sample size (AICc, [47]), through the AICctab function in package *bbmle* [48]. We evaluated the relative importance of the predictor variables using the sum of Akaike weights of all models that included the variable *ω+* [47]. Graphic visualization of the data was implemented using package *plot3D* [49]. All these analyses were conducted in the software R [50].

## 3. Results

### 3.1. Use of Beaches by Dogs

We detected the presence of dogs on 92.9% (*n* = 13) of the beaches, and dog tracks were found on all of them (*n* = 14). At the beach level, we detected evidence of dog presence in 0–80% of the surveyed area when we analyzed direct sightings, and 33–100% when we considered track detection (Table 1). Considering the full extension of the surveyed area (the sum of all 14 beaches), we detected dog presence and dog tracks on at least one occasion in 32% (67/209) and 82% of the segments (171/209), respectively.

Across all samplings, we registered 176 records of dogs and 4349 records of people. The mean number of dogs detected per beach varied between 0.0 ± 0.0 and 11.5 ± 11.3 (Table 1). In the case of people, the mean varied between 2.3 ± 4.5 and 507.5 ± 934.4. Noticeably, we only recorded dogs when people were present on the beach. Supervised dogs represented 13.1% of the animals detected (23/176), whereas the remaining dogs were either unsupervised (44.9%, 79/176) or partially supervised (42.0%, 74/176).

### 3.2. Factors Influencing Dog Abundance

**Figure 2:**
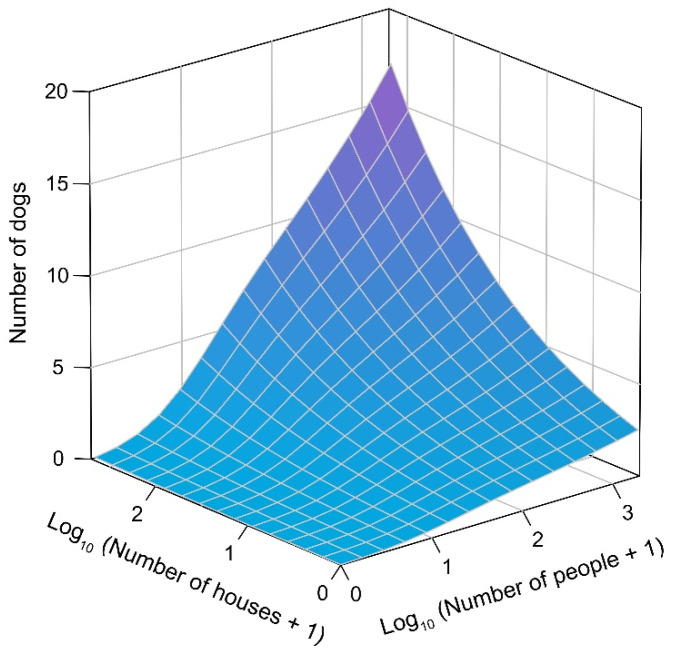
Fitted estimates of number of dogs present at sandy beaches of southern Chile as a function of the number of houses (roofs counted within a 200 m buffer) and number of people present on the beach at the moment of sampling. Note that number of people and number of houses were log-transformed.

The model that better explained the abundance of dogs on beaches included the number of people and the number of houses as predictor variables (Table 2). Both variables were positively associated with the number of dogs recorded (Figure 2). The zero-inflated component of the model shows that the probability of counting zero dogs decreases as the number of people on the beach increases (Table 2). Among the set of candidate variables, we found strong and moderate evidence for the relative importance of the number of houses (*ω*+ = 0.99) and the number of people (*ω*+ = 0.71), respectively. The relative importance of the time of the sampling (*ω*+ = 0.55), day of the sampling (*ω*+ = 0.47), and beach length (*ω*+ = 0.44) was limited.

### 3.3. Dog Harassment of Whimbrels

The mean number of whimbrels detected per beach varied between 0.8 ± 0.5 and 47.3 ± 14.3. Mean density was 6.3 ± 4.6 whimbrel/km, ranging between 0.6 ± 0.4 (Los Molinos) and 13.1 ± 3.5 whimbrels/km (Alepue). We recorded at least one harassment event in 19.6% of the sampling occasions (11/56). At the transect level, we detected 27 events of both dogs and whimbrels co-occurring in a single segment, involving 60 dogs and 40 whimbrels. In 59.3% (n = 16) of these cases, we detected dog harassment of whimbrels. Harassment involved a median of 1.0 dog (range: 1–6) and 1.0 whimbrel (range: 1–3) per event. Among dogs that co-occurred with whimbrels, half (50%) were detected harassing the birds. This corresponds to 17% of all dogs detected on the beaches. Dogs that harassed birds included partially supervised (53.3%, *n* = 16) and unsupervised dogs (46.7%, *n* = 14). Out of the 40 whimbrels that co-occurred with dogs, 60% were harassed (3.8% of the whimbrels recorded). In all cases, whimbrels flew, moving to another area of the same beach. We did not record cases of birds killed or injured.

## 4. Discussion

In this study, we detected the presence of dogs or their tracks on all beaches surveyed. These findings deserve attention considering that these canids are considered an important threat to the conservation of shorebird populations within the Pacific Americas Flyway [51]. We acknowledge that our estimate may be positively biased due to the fact that dog abundance was positively associated with people, strongly suggesting that more isolated beaches could have a better situation (no dogs). However, sampled sites included the majority of the sandy beach ecosystems of the study area and, as a consequence, our results confirm that dogs are present on most of them. Furthermore, dogs use an important proportion of each of the beaches and areas free of dogs were scarce. Based on the evidence presented, we can ascertain that the presence of dogs at sandy beaches of southern Chile is generalized, as reported in other areas of the world [52,53,54].

As expected, human presence was the main driver of dog abundance at sandy beaches, whereas beach length had no effect. When few or no people were present, we rarely detected dogs, and the number of dogs and people was positively associated. This association suggests that an important proportion of dogs may be owned by the visitors and taken to the beach as part of recreational activities [21]. Supporting this assumption, more than half of the dogs detected were supervised or partially supervised, while only 13% corresponded to animals on a leash, similar to previous reports in an urban area of central Chile (16.8%, [41]). Although Chilean legislation does not regulate the use of leashes in public spaces (except for dogs classified as potentially dangerous [55]), some local regulations require their use (in the case of our study area, Municipal Ordinances (e.g., [35])). Therefore, our findings show that most dog owners that visit beaches do not comply with local regulations, similar to the situations at beaches of other countries (e.g., 81% of dogs unleashed in the United States [56]).

In addition to dogs brought to beaches by their owners, nearly 45% of the dogs were unsupervised, which suggests that additional factors determine the presence of dogs at sandy beaches. The positive association between the number of houses (and people) and dog abundance resembles the findings of previous studies in other ecosystems [25] and suggests that a proportion of these animals may belong to properties located near the beach. This is likely, considering that in rural areas of the region, >85% of the households that own dogs allow them to roam without restriction at least occasionally [33,57]. This is a relevant finding, given that most previous research on the topic has been conducted in developed countries characterized by adequate management of pets [16]. The lack of movement restriction of dogs is a generalized problem in Chile, which has been reported in urban areas as a public health problem [41,58] and in rural areas as a problem that affects wildlife conservation [24], livestock husbandry, and human security [33,59]. Furthermore, some of the dogs detected could be abandoned animals, a frequent phenomenon in rural areas of southern Chile [60]. Some of these dogs are adopted by local communities [60], but an unknown proportion stay in natural areas. Coastal areas easily provide resources for these dogs, mainly through human activity (garbage, food intentionally provided, leftovers from fishing activities, etc.) and, to a lesser extent, natural processes, including the stranding of marine animals and predation on marine species that use the coast [61]. However, the fact that dogs were strongly associated with human features, but not with beach length, strongly suggests that the presence of dogs at beaches is mainly driven by human activity rather than by environmental factors.

We did not attempt to evaluate the effects of dogs on birds inhabiting sandy beaches. Nevertheless, our data suggest that when dogs and shorebirds—whimbrels in this case—co-occur, the probability of dogs harassing birds is very high (>50%). Up to 17% of the dogs recorded were observed harassing whimbrels, higher than previous reports (9%, [22]). The proportion of whimbrels harassed by dogs represents a small proportion of the birds detected (3.8%), but this is likely a major underestimation influenced by the small amount of time allocated to observation within each segment. In light of the high proportion of beaches used by dogs, as revealed by the track records, coupled with the behavior of whimbrels maintaining their foraging territories at each beach at nonbreeding areas (e.g., [62]), the proportion of birds harassed by dogs on a daily basis would be much higher than the numbers we report. In addition, whimbrel density within the study area was higher than that reported throughout sandy beaches at different areas of the Pacific Flyway [63,64], reaching similarly high values found at most relevant nonbreeding grounds for the species on Chiloé Island [64]. High densities overall indicate high-quality areas used by whimbrels throughout five months, thus reinforcing the importance of dog management at these sandy beaches in southern Chile for the species. Furthermore, we cannot discard the occurrence of occasional predation events, such as those reported for marine turtle nests [12] and other coastal birds [32]. The widespread presence of unleashed dogs at beaches coupled with a high probability of harassment of whimbrels deserves further research to determine if these interactions—experienced in nonbreeding areas—can lead to negative impacts on this species of high concern in the Americas [65,66], as well as on other species such as oystercatchers (*Haematopus* spp.) and other long-distance migratory shorebirds (e.g., *Calidris alba*).

Our work reveals the need to improve regulations associated with dog management, as well as the enforcement of the existing ones. In Chile, dog owners have the obligation to keep dogs within their property [34], but, as previous studies have shown [33,57], a high proportion of dog owners do not comply with this. Our study also reveals the importance of regulating the use of leashes in public places. Mandatory leash-use was considered in Chile [67]; however, it was not included in the final version of the regulation associated with the responsible ownership law [55]. Nevertheless, the existence of regulation by itself does not guarantee behavioral changes in society. In fact, evidence from other areas of the world suggests that even when there are leash laws, these are not followed by an important proportion of visitors to natural areas [15,54,68,69]. Therefore, we follow Miller et al. [70] in suggesting that a better understanding of the factors that drive dog-management decisions among owners is fundamental to reduce the impact of dogs on natural ecosystems. Strategies such as the use of social marketing [71] could be explored in Chile to improve the management of dogs at sandy beaches and other ecosystems.

## 5. Conclusions

The results of this study show that dog abundance at sandy beaches is positively associated with the number of people on the beach and the number of houses in surrounding areas. The association between dog abundance and number of people on the beach is assumedly due to both dogs being taken to the beach by their owners and free-ranging dogs associating with people. Unfortunately, only a small proportion of dogs (13%) were leashed, and unleashed dogs harassed shorebirds when they were present in the same area of the beach. In light of these results, we suggest that better regulation of dogs roaming in public areas is urgently needed, as well as enforcement of existing laws. Future research should address (1) the impacts of dogs on shorebird populations along the South American Pacific coast, as well as (2) responsible ownership strategies aiming at reducing the number of dogs roaming without supervision.

## Figures and Tables

**Table 1:** Presence and abundance of dogs at sandy beaches of Los Ríos region, southern Chile. The table shows the length of the beach, area used by dogs (percentage of the segments where dogs were sighted or their tracks detected on at least one occasion), the mean number of dogs detected and its standard deviation (SD), the median, and the minimum and maximum number of dogs detected on the beach (Range). Totals correspond to the sum of the extension of the beaches sampled and the proportion of the segments where dogs were detected at least once. We did not include the total for number of dogs to prevent double counting.

Beach	Length (km)	Area Used by Dogs (%)		Number of Dogs Detected per Beach		
		Sightings	Tracks	Mean (SD)	Median	Range
Ronca	0.6	17	33	0.5 (1.0)	0.0	0–2
Cheuque	0.8	75	100	3.5 (3.0)	4.0	0–6
Pichicuyín	0.3	75	100	1.3 (2.5)	0.0	0–5
Mehuín	1.9	68	100	11.5 (11.3)	9.5	0–27
Epuco	1.9	0	37	0.0 (0.0)	0.0	0–0
Alepue	2.3	9	100	1.0 (1.2)	1.0	0–2
Pilolcura	0.6	57	86	1.5 (1.9)	1.0	0–4
Curiñanco	4.7	23	87	5.3 (3.0)	6.0	1–8
Calfuco	1.2	17	100	1.3 (1.5)	1.0	0–3
SanIgnacio	0.8	22	78	1.8 (3.5)	0.0	0–7
LosMolinos	1.2	75	100	6.5 (5.2)	7.0	0–12
Niebla	1.0	80	90	8.3 (4.7)	6.5	5–15
Mancera	1.3	38	92	1.5 (3.0)	0.0	0–6
Chaihuín	2.0	5	45	0.3 (0.5)	0.0	0–1
**Total**	**20.6**	**32**	**82**	**-**	**-**	**-**

**Table 2:** Models that explain the number of dogs detected at sandy beaches of southern Chile. Candidate models with little support (*ω* < 0.05) are not shown. The data presented include the number of parameters (*k*), delta Akaike Information Criterion corrected for small sample size (AICc, ΔAICc), Akaike weight (*ω*), and the parameter estimates and standard errors (in parentheses) for the effects of number of people on the beach (People), number of houses in surrounding areas (Houses), day (Day, week day as reference), time (Time, morning as reference), and beach length (Length) on the Poisson component of the model, and the effect of number of people on the zero-inflated component of the model (Zero-inflated). The random effect variance (Random effects) and its standard deviation in parentheses are also shown.

Model	*k*	ΔAICc	*ω*	People	Houses	Day	Time	Length	Zero- Inflated	Random Effects
People + houses	6	0.0	0.17	0.5 (0.1)	0.7 (0.2)	-	-	-	−2.9 (0.9)	0.1 (0.3)
People + houses + length	7	0.1	0.16	0.4 (0.1)	0.8 (0.2)	-	-	−0.2 (0.1)	−2.9 (0.9)	0.1 (0.2)
Houses + day + time	7	0.2	0.15	-	1.0 (0.2)	0.5 (0.1)	0.5 (0.2)	-	−3.0 (0.9)	0.1 (0.3)
Houses + day + time + length	8	1.1	0.10	-	1.1 (0.2)	0.5 (0.2)	0.5 (0.2)	−0.2 (0.1)	−3.0 (0.9)	0.1 (0.3)
People + houses + time	7	1.1	0.09	0.4 (0.1)	0.7 (0.2)	-	0.2 (0.2)	-	−2.9 (0.9)	0.1 (0.3)
People + houses + time + length	8	1.6	0.08	0.4 (0.1)	0.9 (0.2)	-	0.2 (0.2)	−0.2 (0.1)	−2.9 (0.9)	0.1 (0.2)
People + houses + day + time	8	1.9	0.06	0.2 (0.2)	0.9 (0.3)	0.3 (0.2)	0.4 (0.2)	-	−2.9 (0.9)	0.1 (0.3)
People + houses + day	7	2.2	0.06	0.4 (0.2)	0.8 (0.2)	0.1 (0.2)	-	-	−2.9 (0.9)	0.1 (0.3)
People + houses + day + length	8	2.5	0.05	0.4 (0.2)	0.9 (0.2)	0.1 (0.2)	-	−0.2 (0.1)	−2.9 (0.9)	0.1 (0.2)

## Data Availability

The data presented in this study are available on request from the corresponding author.

## References

[1] McLachlan A., Defeo O. (2017). The Ecology of Sandy Shores.

[2] Schlacher T.A., Schoeman D.S., Dugan J., Lastra M., Jones A., Scapini F., McLachlan A. (2008). Sandy beach ecosystems: Key features, sampling issues, management challenges and climate change impacts. Mar. Ecol..

[3] Barbier E., Hacker S., Kennedy C., Koch E., Stier A., Silliman B. (2011). The value of estuarine and coastal ecosystem services. Ecol. Monogr..

[4] Maguire G.S., Miller K.K., Weston M.A., Young K. (2011). Being beside the seaside: Beach use and preferences among coastal residents of south-eastern Australia. Ocean. Coast. Manag..

[5] Defeo O., McLachlan A., Schoeman D.S., Schlacher T.A., Dugan J., Jones A., Lastra M., Scapini F. (2009). Threats to sandy beach ecosystems: A review. Estuar. Coast. Shelf Sci..

[6] Schlacher T.A., Thompson L.M.C. (2007). Exposure of fauna to off-road vehicle (ORV) traffic on sandy beaches. Coast. Manag..

[7] Dugan J.E., Hubbard D.M., McCrary M.D., Pierson M.O. (2003). The response of macrofauna communities and shorebirds to macrophyte wrack subsidies on exposed sandy beaches of southern California. Estuar. Coast. Shelf Sci..

[8] Duarte C., Quintanilla-Ahumada D., Anguita C., Manríquez P.H., Widdicombe S., Pulgar J., Silva-Rodríguez E.A., Miranda C., Manríquez K., Quijón P.A. (2019). Artificial light pollution at night (ALAN) disrupts the distribution and circadian rhythm of a sandy beach isopod. Environ. Pollut..

[9] Gompper M.E., Gompper M.E. (2014). The dog–human–wildlife interface: Assessing the scope of the problem. Free-Ranging Dogs and Wildlife Conservation.

[10] Bellard C., Genovesi P., Jeschke J.M. (2016). Global patterns in vertebrates threatened by biological invasions. Proc. R. Soc. B Biol. Sci..

[11] Doherty T.S., Dickman C.R., Glen A.S., Newsome T.M., Nimmo D.G., Ritchie E.G., Vanak A.T., Wirsing A.J. (2017). The global impacts of domestic dogs on threatened vertebrates. Biol. Conserv..

[12] Ruiz-Izaguirre E., van Woersem A., Eilers K.C.H.A.M., van Wieren S.E., Bosch G., van der Zijpp A.J., de Boer I.J.M. (2015). Roaming characteristics and feeding practices of village dogs scavenging sea-turtle nests. Anim. Conserv..

[13] Baudains T.P., Lloyd P. (2007). Habituation and habitat changes can moderate the impacts of human disturbance on shorebird breeding performance. Anim. Conserv..

[14] Lord A., Waas J.R., Innes J., Whittingham M.J. (2001). Effects of human approaches to nests of northern New Zealand dotterels. Biol. Conserv..

[15] Weston M.A., Stankowich T., Gompper M.E. (2014). Dogs as agents of disturbance. Free-Ranging Dogs and Wildlife Conservation.

[16] Weston M.A., Fitzsimons J.A., Wescott G., Miller K.K., Ekanayake K.B., Schneider T. (2014). Bark in the park: A review of domestic dogs in parks. Environ. Manag..

[17] Weston M.A., Elgar M.A. (2007). Responses of Incubating Hooded Plovers (*Thinornis rubricollis*) to Disturbance. J. Coast. Res..

[18] Navedo J.G., Verdugo C., Rodríguez-Jorquera I.A., Abad-Gómez J.M., Suazo C.G., Castañeda L.E., Araya V., Ruiz J., Gutiérrez J.S. (2019). Assessing the effects of human activities on the foraging opportunities of migratory shorebirds in Austral high-latitude bays. PLoS ONE.

[19] Lafferty K.D. (2001). Disturbance to wintering western snowy plovers. Biol. Conserv..

[20] Pearce-Higgins J.W., Brown D.J., Douglas D.J.T., Alves J.A., Bellio M., Bocher P., Buchanan G.M., Clay R.P., Conklin J., Crockford N. (2017). A global threats overview for Numeniini populations: Synthesising expert knowledge for a group of declining migratory birds. Bird Conserv. Int..

[21] Guinness S.J., Maguire G.S., Miller K.K., Weston M.A. (2020). My dog, my beach! Attitudes towards dog management on Victorian beaches. Australas. J. Environ. Manag..

[22] Lafferty K.D. (2001). Birds at a Southern California beach: Seasonality, habitat use and disturbance by human activity. Biodivers. Conserv..

[23] Guillén J., García-Olivares A., Ojeda E., Osorio A., Chic O., González R. (2008). Long-Term Quantification of Beach Users Using Video Monitoring. J. Coast. Res..

[24] Silva-Rodríguez E.A., Sieving K.E. (2012). Domestic dogs shape the landscape-scale distribution of a threatened forest ungulate. Biol. Conserv..

[25] Ribeiro F.S., Nichols E., Morato R.G., Metzger J.P., Pardini R. (2019). Disturbance or propagule pressure? Unravelling the drivers and mapping the intensity of invasion of free-ranging dogs across the Atlantic forest hotspot. Divers. Distrib..

[26] Daniels T.J., Bekoff M. (1989). Population and Social Biology of Free-Ranging Dogs, *Canis familiaris*. J. Mammal..

[27] Sepúlveda M., Pelican K., Cross P., Eguren A., Singer R. (2015). Fine-scale movements of rural free-ranging dogs in conservation areas in the temperate rainforest of the coastal range of southern Chile. Mamm. Biol..

[28] Morin D.J., Lesmeister D.B., Nielsen C.K., Schauber E.M. (2018). The truth about cats and dogs: Landscape composition and human occupation mediate the distribution and potential impact of non-native carnivores. Glob. Ecol. Conserv..

[29] Dos Santos C.L., Le Pendu Y., Giné G.A.F., Dickman C., Newsome T.M., Cassano C.R. (2018). Human behaviors determine the direct and indirect impacts of free-ranging dogs on wildlife. J. Mammal..

[30] Corti P., Wittmer H.U., Festa-Bianchet M. (2010). Dynamics of a small population of endangered huemul deer (*Hippocamelus bisulcus*) in Chilean Patagonia. J. Mammal..

[31] Moreira-Arce D., Vergara P.M., Boutin S. (2015). Diurnal Human Activity and Introduced Species Affect Occurrence of Carnivores in a Human-Dominated Landscape. PLoS ONE.

[32] Bravo-Naranjo V., Jiménez R.R., Zuleta C., Rau J.R., Valladares P., Piñones C. (2019). Selección de presas por perros callejeros en el humedal Estero Culebrón (Coquimbo, Chile). Gayana.

[33] Villatoro F.J., Naughton-Treves L., Sepúlveda M.A., Stowhas P., Mardones F.O., Silva-Rodríguez E.A. (2019). When free-ranging dogs threaten wildlife: Public attitudes toward management strategies in southern Chile. J. Environ. Manag..

[34] Ministerio de Salud Ley Núm 21.020: Sobre Tenencia Responsable de Mascotas y Animales de Compañía. https://www.leychile.cl/Navegar?idNorma=1106037.

[35] Municipalidad de Valdivia Aprueba Ordenanza Para la Protección y Control de la Población Canina en la Ciudad de Valdivia. https://www.leychile.cl/Navegar?idNorma=227550.

[36] Jaramillo E., McLachlan A. (1993). Community and Population Responses of the Macroinfauna to Physical Factors over a Range of Exposed Sandy in South-central Chile. Estuar. Coast. Shelf Sci..

[37] Duarte C., Jaramillo E., Contreras H., Acuña K., Navarro J.M. (2009). Importancia del subsidio de macroalgas sobre la abundancia y biología poblacional del anfípodo *Orchestoidea tuberculata* (Nicolet) en playas arenosas del centro sur de Chile. Rev. Biol. Mar. Oceanogr..

[38] Aparicio A. (2002). Calidad de Hábitat en Playas Arenosas del Centro sur de Chile para aves Playeras Migratorias: Análisis de su Importancia como áreas de Parada. Ph.D. Thesis.

[39] BCN (Biblioteca del Congreso Nacional de Chile) Mapas Vectoriales. https://www.bcn.cl/.

[40] Burger J., Niles L. (2013). Shorebirds and stakeholders: Effects of beach closure and human activities on shorebirds at a New Jersey coastal beach. Urban. Ecosyst..

[41] Ibarra L., Espínola F., Echeverría M. (2006). Una prospección a la población de perros existente en las calles de la ciudad de Santiago, Chile. Av. En Ciencias Vet..

[42] Fonseca J., Basso E., Serrano D., Navedo J.G. (2017). Effects of tidal cycles on shorebird distribution and foraging behaviour in a coastal tropical wetland: Insights for carrying capacity assessment. Estuar. Coast. Shelf Sci..

[43] Banks P.B., Bryant J.V. (2007). Four-legged friend or foe? Dog walking displaces native birds from natural areas. Biol. Lett..

[44] Zuur A.F., Ieno E.N., Walker N., Saveliev A., Smith G. (2009). Mixed Effects Models and Extensions in Ecology with R.

[45] Zuur A.F., Ieno E.N., Elphick C.S. (2010). A protocol for data exploration to avoid common statistical problems. Methods Ecol. Evol..

[46] Brooks M.E., Kristensen K., van Benthem K.J., Magnusson A., Berg C.W., Nielsen A., Skaug H.J., Mächler M., Bolker B.M. (2017). glmmTMB balances speed and flexibility among packages for zero-inflated generalized linear mixed modeling. R J..

[47] Burnham K.P., Anderson D.R. (2002). Model Selection and Inference: A Practical Information-Theoretic Approach.

[48] Bolker B.M., R. Core Team bbmle: Tools for General Maximum Likelihood Estimation. R Package Version 1.0.23.1. https://cran.r-project.org/package=bbmle.

[49] Soetaert K. plot3D: Plotting Multi-Dimensional Data. R Package Version 1.3. https://cran.r-project.org/package=plot3D.

[50] R Core Team (2020). R: A Language and Environment for Statistical Computing.

[51] Senner S.E., Andres B.A., Gates H.R. (2016). Pacific Americas Shorebird Conservation Strategy.

[52] Stigner M.G., Beyer H.L., Klein C.J., Fuller R.A. (2016). Reconciling recreational use and conservation values in a coastal protected area. J. Appl. Ecol..

[53] Maguire G.S., Miller K.K., Weston M.A., Makowski C., Finkl C.W. (2019). Only the Strictest Rules Apply: Investigating Regulation Compliance of Beaches to Minimize Invasive Dog Impacts on Threatened Shorebird Populations. Impacts of Invasive Species on Coastal Environments: Coasts in Crisis.

[54] Schneider T.J., Maguire G.S., Whisson D.A., Weston M.A. (2019). Regulations fail to constrain dog space use in threatened species beach habitats. J. Environ. Plan. Manag..

[55] SUBDERE Reglamento que Establece la Forma y Condiciones en Que se Aplicarán las Normas Sobre Tenencia Responsable de Mascotas y Animales de Compañía y Determina Las Normas Que Permitirán Calificar a Ciertos Especímenes Caninos Como Potencialmente Peligrosos. https://www.leychile.cl/Navegar?idNorma=1121980.

[56] Lafferty K.D., Rodriguez D.A., Chapman A. (2013). Temporal and spatial variation in bird and human use of beaches in southern California. Springer Plus.

[57] Sepúlveda M.A., Singer R.S., Silva-Rodriǵuez E., Stowhas P., Pelican K. (2014). Domestic dogs in rural communities around protected areas: Conservation problem or conflict solution?. PLoS ONE.

[58] Astorga F., Escobar L.E., Poo-Muñoz D.A., Medina-Vogel G. (2015). Dog ownership, abundance and potential for bat-borne rabies spillover in Chile. Prev. Vet. Med..

[59] Montecino-Latorre D., San Martín W. (2019). Evidence supporting that human-subsidized free-ranging dogs are the main cause of animal losses in small-scale farms in Chile. Ambio.

[60] Villatoro F.J., Sepúlveda M.A., Stowhas P., Silva-Rodríguez E.A. (2016). Urban dogs in rural areas: Human-mediated movement defines dog populations in southern Chile. Prev. Vet. Med..

[61] Pavés H., Schlatter R., Espinoza C. (2008). Scavenging and predation by Black Vultures *Coragyps atratus* at a South American sea lion breeding colony. Vulture News.

[62] Johnston-González R., Abril E. (2019). Predation risk and resource availability explain roost locations of Whimbrel *Numenius phaeopus* in a tropical mangrove delta. IBIS.

[63] Neuman K.K., Henkel L.A., Page G.W. (2008). Shorebird Use of Sandy Beaches in Central California. Waterbirds.

[64] Andres B.A., Johnson J.A., Valenzuela J., Morrison R.I.G., Espinosa L.A., Ross R.K. (2009). Estimating Eastern Pacific Coast Populations of Whimbrels and Hudsonian Godwits, with an Emphasis on Chiloé Island, Chile. Waterbirds.

[65] Brown S., Hickey C., Harrington B., Gill R. (2001). The U.S. Shorebird Conservation Plan.

[66] Donaldson G.M., Hyslop C., Morrison R.I.G., Davidson I. (2000). Canadian Shorebird Conservation Plan.

[67] SUBDERE Reglamento Sobre Tenencia Responsable de Mascotas y Animales de Compañía. http://www.subdere.gov.cl/sites/default/files/version_final_para_consulta_publica.pdf.

[68] Thomas K., Kvitek R.G., Bretz C. (2002). Effects of human activity on the foraging behavior of sanderlings *Calidris alba*. Biol. Conserv..

[69] Parsons A.W., Bland C., Forrester T., Baker-Whatton M.C., Schuttler S.G., McShea W.J., Costello R., Kays R. (2016). The ecological impact of humans and dogs on wildlife in protected areas in eastern North America. Biol. Conserv..

[70] Miller K.K., Ritchie E.G., Weston M.A., Gompper M.E. (2014). The human dimensions of dog-wildlife interactions. Free-Ranging Dogs and Wildlife Conservation.

[71] Smith R.J., Verissimo D., Macmillan D.C., Leader-Williams N., Adams W.M., Smith R.J. (2010). Marketing and Conservation: How to Lose Friends and Influence People. Trade-Offs in Conservation: Deciding What to Save.

